# Association between older age and outcome after cardiac surgery: a population-based cohort study

**DOI:** 10.1186/s13019-014-0177-6

**Published:** 2014-11-18

**Authors:** Wei Wang, Sean M Bagshaw, Colleen M Norris, Rami Zibdawi, Mohamad Zibdawi, Roderick MacArthur, for the APPROACH Investigators

**Affiliations:** Division of Cardiac Surgery, Mazankowski Alberta Heart Institute, University of Alberta, Edmonton, Alberta Canada; Division of Critical Care Medicine, Faculty of Medicine and Dentistry, University of Alberta, Edmonton, Alberta Canada; Faculty of Medicine and Dentistry, University of Alberta, Edmonton, Alberta Canada; Faculty of Nursing, University of Alberta, Edmonton, Alberta Canada; Division of Critical Care Medicine, Clinical Sciences Building, 2-124E, 8440 - 112 Street, Edmonton, T6G 2B7 Alberta Canada

**Keywords:** Octogenarian, Cardiac Surgery, Complication, Mortality

## Abstract

**Objective:**

Octogenarians (aged ≥ 80 years) are increasingly being referred for cardiac surgery. We aimed to describe the morbidity, mortality, and health services utilization of octogenarians undergoing elective cardiac surgery.

**Methods:**

Retrospective population-based cohort study of adult patients receiving elective cardiac surgery between January 1 2004 and December 31 2009. Primary exposure was age ≥80 years. Outcomes were 30-day, 1- and 5-year mortality, post-operative complications, and ICU/hospital lengths of stay. Multi-variable logistic and Cox regression analyses were used to explore the association between older age and outcome.

**Results:**

Of 6,843 patients receiving cardiac surgery, 544 (7.9%) were octogenarians. There was an increasing trend in the proportion of octogenarians undergoing surgery during the study period (0.3% per year, P = 0.073). Octogenarians were more likely to have combined procedures (valve plus coronary artery bypass or multiple valves) compared with younger strata (p < 0.001). Crude 30-day, 1-year and 5-year mortality for octogenarians were 3.7%, 10.8% and 29.0%, respectively. Compared to younger strata, octogenarians had higher adjusted 30-day (OR 4.83, 95%CI 1.30-17.92; P = 0.018) and 1-year mortality (OR 4.92; 95% CI, 2.32-10.46. P<0.001). Post-operative complications were more likely among octogenarians. Octogenarians had longer post-operative stays in ICU and hospital, and higher rates of ICU readmission (P < 0.001 for all). After multi-variable adjustment, age ≧ 80 years was an independent predictor of death at 30-days and 1 year.

**Conclusions:**

Octogenarians are increasingly referred for elective cardiac surgery with more combined procedures. Compared to younger patients, octogenarians have a higher risk of post-operative complications, consume greater resources, and have worse but acceptable short and long-term survival.

**Electronic supplementary material:**

The online version of this article (doi:10.1186/s13019-014-0177-6) contains supplementary material, which is available to authorized users.

## Background

Population aging is a global phenomenon with significant implications for health care systems in the developed world. In 2012, over 5.2 million people in Canada were aged over 65 years, representing 14.9% of total population. Of these, approximately 1.4 million (4.2% of total population) were octogenarians (aged ≥80 years) [1]. Associated with this increasingly older population, the demand for surgery, in particular cardiac surgery, is expected to markedly increase [2],[3].

Prior studies evaluating the course and outcomes of older patient receiving cardiac surgery have notable limitations, including being relatively small, only investigating certain type or types of cardiac procedures, failing to include adjusted analyses of outcomes relative to younger age strata, and reporting only on short-term outcomes [4]-[7].

We hypothesized that octogenarians undergoing cardiac surgery would have higher perioperative mortality and complication rates and consume greater health resources. Our objectives were to describe the population-based short-term and long-term mortality, major morbidity, and health services utilization in patients aged ≥80 years undergoing elective cardiac surgery.

## Methods

The study was approved by the Health Research Ethics Board at the University of Alberta prior to commencement. The requirement for written consent was waived.

### Study design, setting, population

This was a retrospective population-based cohort study performed at the Mazankowski Alberta Heart Institute (MAHI), University of Alberta (Edmonton, Canada) between January 1 2004 and December 31 2009. The MAHI is the only referral center for adult cardiac surgery in northern Alberta. There are nine adult cardiac surgeons at the MAHI performing approximately 1100–1200 adult open cardiac surgery procedures annually (excluding heart transplant, lung transplant, and mechanical assist device implantation).

All adult patients receiving elective cardiac surgery were potentially eligible. We excluded those patients receiving heart and lung transplantation, emergent cardiac surgery, mechanical assist device implantation and those aged less than 18 years. Octogenarians were defined by an age ≥80 years according to their age on the day of surgery. For the purpose of analyses, the entire cohort was categorized into four age strata: 18–49 years, 50–64 years, 65–79 and ≥80 years, respectively.

### Study protocol

Patients were identified by interrogation of the Alberta Provincial Project for Outcome Assessment in Coronary Heart Disease (APPROACH)) registry. The APPROACH registry prospectively captures detailed clinical information on adult patients with known or suspected coronary artery disease (CAD) investigated in the province of Alberta (more information can be found at http://www.approach.org/). Patients entered into the registry are longitudinally followed after cardiac catheterization for all cardiac-specific investigations, interventions (i.e. percutaneous coronary intervention [PCI] or coronary artery bypass grafting surgery [CABG]) and outcomes. Data elements in the registry include socio-demographic features (i.e. age, sex), cardiovascular risk factors and co-morbidities (i.e. hypertension, dyslipidemia, stroke, congestive heart failure (CHF), chronic pulmonary disease, end stage kidney disease (ESKD) on dialysis, peripheral vascular disease (PVD), diabetes mellitus, liver and gastrointestinal disease, malignancy), coronary anatomy as defined by the Duke Index [8], left ventricular ejection fraction (EF), prior thrombolytic therapy, prior myocardial infarction (MI), prior CABG surgery, and prior angioplasty. In addition, the registry also captures several peri-operative variables specific for cardiac surgery (i.e. procedure type, cardiopulmonary bypass and aortic cross clamp time, use of intra-operative balloon pump [IABP]) and surgery specific complications and outcomes. Quarterly merges of the APPROACH registry with the Bureau of Vital Statistics was used for ascertainment of vital status.

### Operational definitions

*Post-operative major bleeding* was defined as blood loss from chest tubes greater than 400 ml × 1 hr or 300 ml × 2 hrs or 200 ml × 3 hrs. *Surgical wound infection* was defined as purulent discharge from sternal wounds and/or positive culture results from blood, urine, sputum/lungs and incisions. *Cardiac arrest* was defined as a recorded cardiac arrest or pulseless electric arrest (PEA) in the post-operative period that required cardiopulmonary resuscitation. *Heart block* was defined as second degree type 2 or third degree heart block occurring beyond post-operative day 3. *Pulmonary complication* was defined as post-operative ventilation time greater than 48 hours or need for re-intubation. *Neurologic complication* was defined as post-operative transient ischemic attack (TIA), stroke or seizure. *Acute kidney injury (AKI)* was defined as an increase of ≥50% in serum creatinine (Cr) from pre-operative baseline value, or post-operative urine output <0.5 ml/kg/hr x 12 hours.

### Statistical analysis

The primary exposure of interest across all analyses was octogenarians (i.e. age ≥80 years). The outcomes were 30-day, 1-year and 5-year mortality, post-operative complications (i.e. bleeding, infection, neurologic events, pulmonary complications, AKI, atrial fibrillation, cardiac arrest, heart block) and health service utilization (ICU stay, hospital stay and ICU re-admission). Continuous data were reported as means ± standard deviations (SD) and were compared by Student’s t-test and analysis of variance (ANOVA) where appropriate. Categorical data were reported as proportions and compared using χ2 test or Fisher’s exact test where appropriate. Multivariate logistic regression analysis was used to identify independent risk factors for 30-day and 1-year mortality in all patients with age strata. Initial covariates considered included sex, pre-operative co-morbid illnesses, procedure type, CPB and/or aortic cross clamp time, and post-operative complications. Data were presented as adjusted odds ratios (ORs) with 95% confidence intervals (CIs). Kaplan-Meier survival curve was used for survival analysis. Cox regression analysis was used to explore the association of long-term mortality across age strata while controlling for potential confounding covariates. The associations were reported as hazard ratios (HR) with 95% CIs. A p-value of <0.05 was considered statistically significant for all comparisons. All analysis was performed by using IBM SSPS software.

## Results

**Figure 1:**
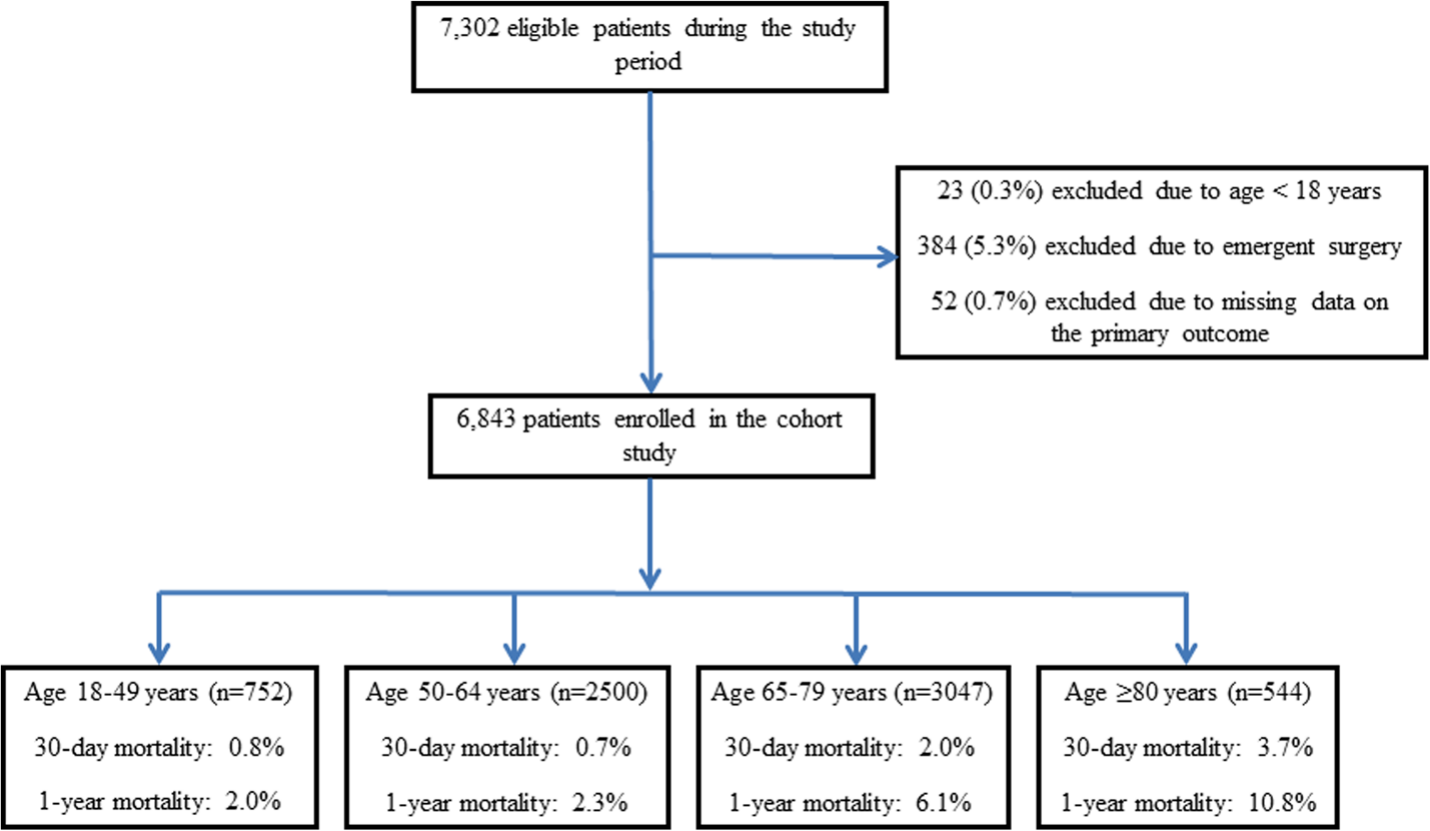
**Flow diagram of study patients.**

**Figure 2:**
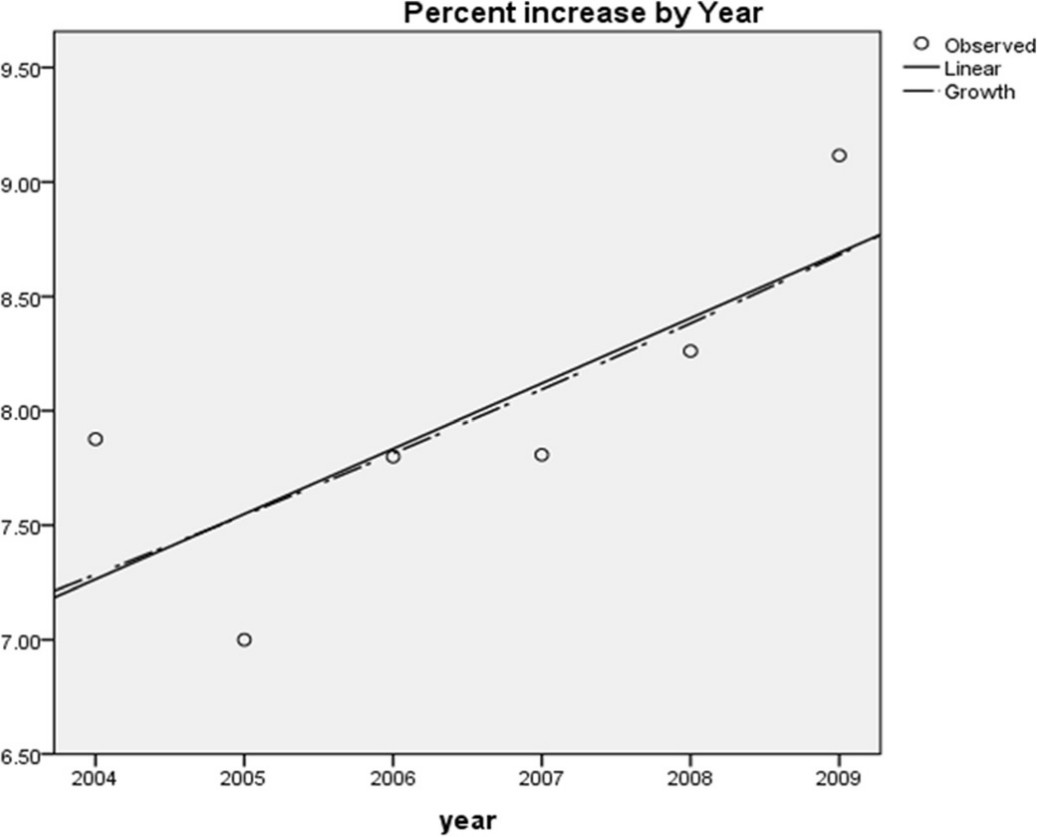
**Annual proportions of octogenarians receiving cardiac surgery within the study period.**

Of 7,302 patients receiving cardiac surgery during the study, 23 (0.3%) were excluded due to age <18 years; 384 (5.3%) excluded due to emergent surgery, and 52 (0.7%) excluded due to missing data on the primary outcome. (Figure 1) The final cohort comprised of 6,843 patients undergoing elective cardiac surgery. Of these, 752 (11.0%) were aged between 18 to 49 years; 2,500 (36.5%) between 50 to 64 years; 3,047 (44.5%) between 65 to 79 years and 544 (7.9%) were aged ≥80 years. The median (range) age of those ≥80 years was 82.5 years (80.0-92.1), with 19.1% (n = 104) ≥85 years and 1.3% (n = 7) ≥90 years, respectively. Linear regression showed a trend for a greater proportion of patients aged ≥80 years receiving cardiac surgery during the study period (estimated relative annual increase 3%; P = 0.073) (Figure 2).

### Baseline characteristics

Octogenarians had higher prevalence of hypertension, prior MI, CHF, stroke, and malignancy when compared with younger strata (Table 1). Octogenarians had lower prevalence of ESKD and had lower BMI values when compared with younger groups. Octogenarians were also less likely to be undergoing re-operation.

**Table 1:** **Pre-operative characteristics and type of surgery across the four age strata**

Variables	Age 18–49 (n = 752)	Age 50–64 (n = 2500)	Age 65–79 (n = 3047)	Age ≥80 (n = 544)	P value
Age (year)	40.6 ± 8.9	58.5 ± 4.1	72.4 ± 4.3	83.1 ± 2.4	<0.001
Male (%)	71.5%	80.9%	74.7%	73.0%	<0.001
Hypertension (%)	42.9%	77.6%	84.3%	86.0%	<0.001
Hyperlipidemia (%)	64.5%	94.9%	94.0%	90.9%	<0.001
Diabetes (%)	14.9%	33.8%	34.0%	26.1%	<0.001
Previous MI (%)	32.3%	50.7%	51.7%	53.8%	<0.001
CHF (%)	12.0%	11.7%	17.8%	24.5%	<0.001
PVD (%)	2.7%	6.7%	11.6%	11.8%	<0.001
Stroke (%)	7.0%	8.9%	16.3%	21.8%	<0.001
CRF on dialysis (%)	2.4%	1.3%	1.5%	0.4%	0.028
Malignancy (%)	1.3%	2.0%	5.6%	7.2%	<0.001
BMI	29.4 ± 5.8	30.1 ± 5.9	28.8 ± 5.1	26.8 ± 4.5	<0.001
Re-operation (%)	21.5%	6.4%	6.9%	4.5%	<0.001
Re-op adult congenital (%)	10.2%	0.3%	0.1%	0.0%	<0.001
Pre-op Cr (μmol/L)	108.1 ± 107.1	103.1 ± 79.0	112.1 ± 83.7	109.3 ± 39.5	0.04
Type of Surgery					
CABG	42.0%	73.2%	67.8%	52.4%	<0.001
AVR	16.0%	5.0%	4.4%	3.9%	<0.001
MVR	15.6%	5.8%	5.3%	4.4%	<0.001
Combined surgery	9.8%	12.6%	21.2%	36.3%	<0.001
AVR + CABG	1.2%	4.6%	11.1%	24.3%	<0.001
MVR + CABG	1.3%	4.1%	5.5%	5.0%	<0.001
2 or more valves	7.3%	3.9%	4.6%	7.0%	<0.001
PVR/TVR	10.6%	1.0%	0.5%	0.0%	<0.001
Others	6.0%	2.3%	0.8%	1.3%	<0.001
CPB time (min)	123.0 ± 62.1	115.9 ± 52.4	123.9 ± 55.2	129.6 ± 55.3	<0.001
Cross Clamp time (min)	79.1 ± 54.9	78.7 ± 44.3	85.8 ± 47.9	92.0 ± 48.4	<0.001
off-pump surgery	9.6%	1.3%	0.9%	0.4%	<0.001

MI: myocardial infarct; CHF: congestive heart failure; PVD: peripheral vascular disease; ESKD: end stage kidney disease; BMI: body mass index; Cr: creatinine, EF: ejection fraction; CABG: coronary artery bypass grafting; AVR: aortic valve repair/replacement; MVR: mitral valve repair/replacement; PVR: pulmonary valve repair/replacement; TVR: tricuspid valve repair/replacement; CPB: cardiopulmonary bypass.

### Surgery type

Octogenarians were more likely to undergo combined cardiac procedures, in particular aortic value replacement and coronary artery bypass, when compared with younger strata. This was associated with comparatively longer durations of CPB and aortic cross-clamp (Table 1).

### Mortality

Crude 30-day, 1-year and 5-year mortality for octogenarians were 3.7%, 10.8% and 29.0%, respectively. The OR of adjusted 30-day and 1-year mortality in octogenarians were 4.83 (95% CI 1.30-17.92, P = 0.018) and 4.92 (95%CI 2.32-10.46, P < 0.001), respectively (referent group 18–49 years).

### Post-operative complications

Octogenarians had significant higher risk for all major post-operative complications (bleeding, infection, neurologic events, pulmonary complications, AKI, atrial fibrillation, cardiac arrest, heart block). Octogenarians also had higher utilization of post-operative continuous renal replacement therapy (CRRT) and a trend for higher utilization of peri-operative IABP (Table 2).

**Table 2:** **Post-operative complications and health service utilization**

Variables	Age 18–49 (n = 752)	Age 50–64 (n = 2500)	Age 65–79 (n = 3047)	Age ≥80 (n = 544)	P value
Bleeding (re-exploration)	2.0%	1.4%	2.3%	2.8%	0.04
Infection	6.2%	7.4%	11.4%	13.4%	<0.001
Neurologic events	1.3%	1.3%	3.7%	7.4%	<0.001
Pulmonary complications	14.1%	12.7%	20.5%	30.3%	<0.001
AKI	6.5%	4.7%	11.2%	16.2%	<0.001
Atrial fibrillation	15.1%	28.0%	41.1%	45.8%	<0.001
Cardiac arrest	1.5%	1.8%	3.4%	5.5%	<0.001
Heart block	2.7%	1.8%	2.9%	5.0%	<0.001
IABP	3.0%	3.1%	3.9%	4.2%	0.27
CRRT	2.7%	1.7%	4.1%	6.4%	<0.001
CVICU LOS (hr)	87.5 ± 385.6	67.7 ± 145.0	107.7 ± 248.7	158.8 ± 343.6	<0.001
Hospital LOS	8.7 ± 14.4	8.1 ± 14.7	11.3 ± 17.7	15.8 ± 24.2	<0.001
CVICU re-admission	3.9%	2.6%	4.6%	9.0%	<0.001
Discharge location					
Home	92.1%	90.6%	77.1%	56.1%	<0.001
Other facilities	7.9%	9.4%	22.9%	43.9%	<0.001

AKI: acute kidney injury; IABP: intra-aortic balloon pump; CRRT: continuous renal replacement therapy; LOS: length of stay.

### Health service utilization

Octogenarians had longer durations of stay in ICU and hospital (Table 2). Octogenarians were also considerably more likely to require ICU readmission. The crude 30-day, 1-year and 5-year mortality in octogenarians who had cardiovascular surgical ICU (CVICU) re-admission were 10.2%, 40.8% and 59.2%. The likelihood of not being discharged home or to a skilled nursing facility was higher for octogenarians (Table 2).

### Factors associated with 30-day, 1-year and 5-year mortality

In multi-variable analysis, age ≥80 years was independently associated with increased risk of death at 30 days (adjusted OR 4.83, 95% CI 1.30-17.92, P = 0.0018) and at 1-year (adjusted OR 4.92, 95% CI 2.32-10.46, P < 0.001) (Tables 3 and 4).

**Table 3:** **Significant independent predictive variables for 30-day mortality by multivariate logistic regression analysis**

Factors	OR (95% CI)	P value
Pre-operative factors		
Male sex	3.00 (1.75-5.15)	<0.001
Age ≥80 (referent group 18–49 years)	4.83 (1.30-17.92)	0.018
ESKD on dialysis	6.54 (2.52-16.94)	<0.001
EF 35-50% (referent group EF > 50%)	2.29 (1.08-4.86)	0.032
EF < 35% (referent group EF > 50%)	4.07 (1.70-9.74)	0.002
Intra-operative factors		
CPB time	1.01 (1.01-1.02)	<0.001
Post-operative complications		
Pulmonary complications	2.39 (1.30-4.41)	0.005
Cardiac arrest	20.20 (10.64-38.34)	<0.001
Acute kidney injury on CRRT	5.13 (2.57-10.21)	<0.001

OR: odds ratio; CI: confidence interval; CRF: chronic renal failure; EF: ejection fraction; CPB: cardiopulmonary bypass; CRRT: continuous renal replacement therapy.

**Table 4:** **Significant independent predictive variables for 1-year mortality by multivariate logistic regression analysis**

Factors	OR (95% CI)	P value
Pre-operative factors		
Age 65–79 (referent group 18–49 years)	3.01 (1.52-5.98)	0.002
Age ≥80 (referent group 18–49 years)	4.92 (2.32-10.46)	<0.001
CHF	2.13 (1.54-2.94)	<0.001
Peripheral vascular disease	2.04 (1.40-2.96)	<0.001
ESKD on dialysis	6.80 (3.68-12.55)	<0.001
EF < 35% (referent group EF > 50%)	1.83 (1.12-3.01)	0.017
Intra-operative factors		
CPB time	1.01 (1.00-1.01)	0.001
Post-operative complications		
Neurologic events	1.82 (1.13-2.93)	0.014
Pulmonary complications	2.14 (1.53-3.00)	<0.001
Cardiac arrest	5.38 (3.36-8.62)	<0.001
CVICU re-admission	1.61 (1.04-2.48)	0.034
Acute kidney injury on CRRT	6.27 (4.03-9.75)	<0.001

OR: odds ratio; CI: confidence interval; CHF: congestive heart failure; CRF: chronic renal failure; EF: ejection fraction; CPB: cardiopulmonary bypass; CRRT: continuous renal replacement therapy; CVICU: cardiovascular surgical intensive care unit.

**Figure 3:**
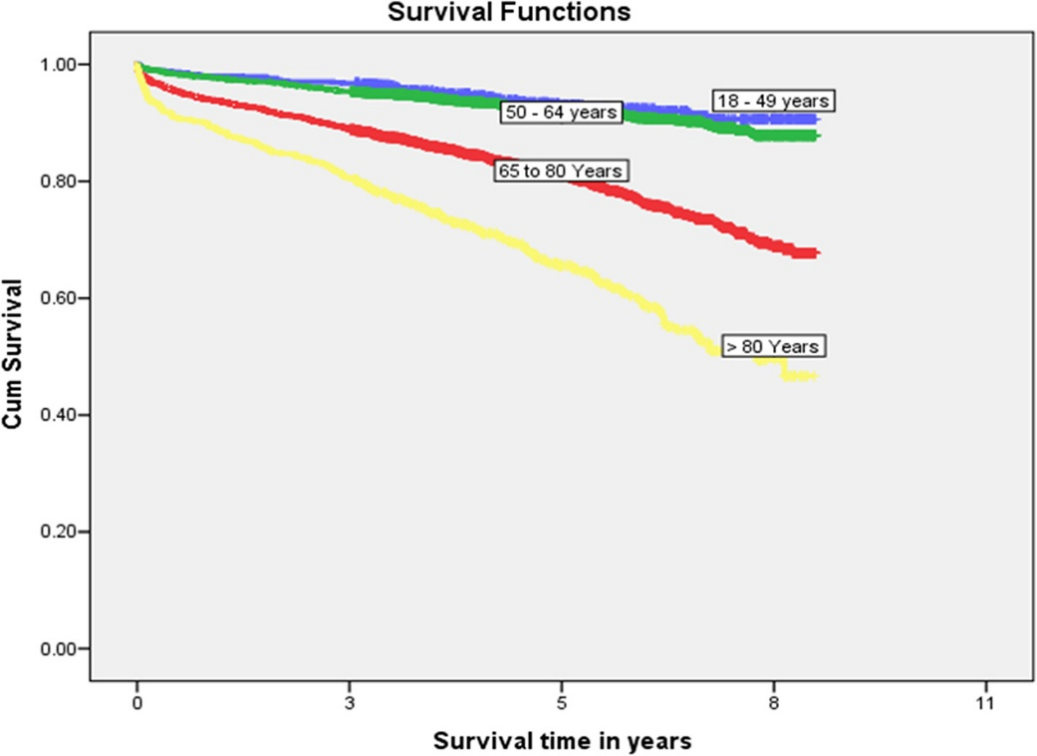
**Kaplan-Meier survival curve for long-term survival.**

Age ≥80 years was the strongest independent factor affecting long-term survival (hazard ratio [HR] 5.30, 95% CI 3.70-7.59, P < 0.001) (Table 5; Figure 3). While procedure type was not independently associated with short or mid-term mortality; those receiving combined procedures had worse long term survival.

**Table 5:** **Significant risk factors for long-term survival by Cox regression analysis**

Factors	HR (95% CI)	P value
Age 65–79 (referent group 18–49 years)	2.81 (2.02-3.90)	<0.001
Age ≥80 (referent group 18–49 years)	5.30 (3.70-7.59)	<0.001
Pre-operative co-morbidities		
Type 1 Diabetes	1.98 (1.17-3.34)	0.011
Type 2 Diabetes	1.43 (1.25-1.64)	<0.001
Smoking	1.52 (1.25-1.86)	<0.001
CHF	1.72 (1.47-2.00)	<0.001
PVD	1.47 (1.24-1.74)	<0.001
Stroke	1.33 (1.14-1.56)	<0.001
ESKD on dialysis	3.61 (2.64-4.94)	<0.001
Malignancy	2.08 (1.66-2.61)	<0.001
EF 35-50% (referent group EF > 50%)	1.20 (1.00-1.43)	0.045
EF < 35% (referent group EF > 50%)	1.54 (1.23-1.94)	<0.001
Surgery type		
AVR + CABG (referent group isolated CABG)	1.61 (1.29-2.00)	<0.001
MVR + CABG (referent group isolated CABG)	1.31 (1.00-1.70)	0.047
2 or more valves (referent group isolated CABG)	1.48 (1.08-2.04)	0.015
Post-operative complications		
Infection	1.28 (1.07-1.53)	0.007
Neurologic events	1.45 (1.12-1.88)	0.004
Pulmonary complications	1.27 (1.08-1.50)	0.004
Acute kidney injury on CRRT	2.10 (1.74-2.54)	<0.001
Cardiac arrest	2.72 (2.07-3.58)	<0.001
CVICU re-admission	1.32 (1.05-1.67)	0.019

HR: hazard ratio; CI: confidence interval; PCI: percutaneous coronary intervention; CHF: congestive heart failure; PVD: peripheral vascular disease; CVA: cerebral vascular attack; CRF: chronic renal failure; EF: ejection fraction; CABG: coronary artery bypass grafting; AVR: aortic valve repair/replacement; MVR: mitral valve repair/replacement; CRRT: continuous renal replacement therapy; CVICU: cardiovascular surgical intensive care unit.

## Discussion

We performed a large population-based cohort study evaluating the incidence, clinical course, outcomes and health utilization of octogenarians undergoing elective cardiac surgery.

### Key findings

Our study had several notable findings. Firstly, the proportion of octogenarians receiving cardiac surgery appears to be increasing. Secondly, octogenarians had significantly higher short and long-term mortality compared with younger patients after adjustment for relevant confounders. Thirdly, octogenarians were more likely to require combined procedures, resulting in longer exposure to cardiopulmonary bypass. Moreover, despite octogenarians having fewer selected comorbid diseases (i.e. ESKD) or undergoing re-operation, the rate of post-operative complications, including ICU readmission, was considerably higher when compared with younger age strata. Finally, octogenarians had markedly longer durations of stay in ICU and hospital and were far less likely to return home after surgery.

### Strengths and limitations

There are important limitations to our study. Firstly, our cohort was assembled from a single center’s experience in Canada, was retrospective, and considered only patients referred for elective surgery. This may limit the generalizability of our study and we recognize the process of selection of octogenarians for elective surgery may be variable within and across institutions and jurisdictions. However, our study utilizes a large catchment from a relatively isolated geographical region of Canada, our cohort was relatively large with complete ascertainment of long-term vital status, and we utilized the high fidelity data prospectively captured with the APPROACH database. Secondly, while we were able to capture hospital discharge disposition and long-term vital status, we were unable to describe quality-adjusted survival or perform more formal cost analyses associated with elective cardiac surgery in octogenarians.

### Interpretation and context with prior literature

Owing to the demographic change worldwide, the relative proportion of older persons is increasing. Data from Australia and New Zealand demonstrated a 5.6% annual increase of octogenarians admitted to ICU [9]. It is not surprising that elderly patients are more commonly referred to cardiac surgery. In fact, the average age for patients undergoing isolated CABG has increased from 55.8 (1990) to 68.6 year (2007) in Germany [10]. In large observational studies, Bhamidipati *et al.* and Alexander *et al.* reported 6.7-7.0% of all patients undergoing cardiac surgeries were aged ≥80 years. In our study cohort, the rate of octogenarians was modestly higher at 7.9%. Precise estimates of the numbers of octogenarians receiving cardiac surgery and their course are vital for resource planning, as our study has clearly shown these patients have longer more complicated post-operative stays that consumer greater resources that will likely impact cardiac surgical capacity and throughput. Although there was no previous data demonstrating the trend of octogenarians undergoing cardiac surgery, our study showed a small but steady annual increase in the number of octogenarians receiving surgery. As aging is a global phenomenon involved both developed and developing countries, we believe this trend of increasing numbers of octogenarians undergoing cardiac surgery would be evidenced by all high volume cardiac surgery centres in the near future.

As a consequence of older patient referral for cardiac surgery, cardiac pathology is often more considerable. Indeed, we found 36.3% of octogenarians received combined cardiac procedures (predominantly AVR and CABG), which is among the highest rates compared to previous studies [10]-[12] and associated with longer CPB and aortic cross clamp time. It is not surprising that octogenarians with more complex procedures have higher risk for major post-operative complications and consequently greater health service utilization (i.e. longer hospital stay, higher ICU re-admission and greater utilization of organ support) and overall costs. Cox regression analysis also suggested combined surgery was associated with worse long term survival. This will present a challenge for cardiac surgical programs to maintain capacity and accommodate the growing demand of this demographic coupled with their higher resource utilization. It is likely that improved and transparent mechanisms for risk assessment and prioritization are needed.

One concern with increasing numbers of octogenarians undergoing more combined cardiac surgery is the high operational mortality. Operating on this specific demographic is not only a challenge to cardiac surgeons but also a challenge to cardiologists, anesthesiologists, CVICU intensivists, and the whole medical system [11]. Previous studies have shown that the in-hospital mortality in octogenarians undergoing isolated CABG, AVR and MVR were 6.8%-14.3% [5],[12]-[14], 4.5%-12.6% [6],[15]-[17], and 2.7%-18.5% [7],[18], respectively. In a multi-center retrospective study gathering data from 22 high volume cardiac centers in the United States, the reported in-hospital mortality rates for octogenarians after cardiac surgery were 8.1% for isolated CABG, 10.1% for combined CABG and AVR, and 19.6% for combined CABG and MVR [19]. In our study, despite more combined cardiac procedures and longer CPB/cross clamp time, the crude 30-day mortality was only 3.7%, and the 1-year and 5-year mortality were 10.8% and 29.0% among octogenarians, respectively. Although still significantly higher than patients of younger age groups, the short-term and long-term survival in octogenarians are better than previous studies. Indeed, the 29.0% 5-year mortality in octogenarians (mean age 83.1 years) undergoing cardiac surgery is lower than the 5-year estimated mortality of an average Canadian at age of 83 years (40.5% in male and 30.4% in female) [20]. These observations confirm that informed selection of older patients for cardiac surgery can translate into acceptable post-operative survival.

Post-operative complications are a source of major morbidity and a driver of health resource use. Across the entire spectrum of complications, the rates are markedly higher among octogenarians [21]. While some occur commonly in the post-operative period, many translate into need for increased intensity of support, downstream morbidity and disability. Indeed, post-operative complications contribute to higher ICU readmission rates and longer durations of ICU and hospital stay. For those octogenarians requiring ICU readmission, in hospital death was 35% and only 41% were discharged home. In addition, post-operative complications were independently associated with 30-day and 1-year mortality.

In our opinion, thoughtful patient selection is necessary to optimize post-operative outcomes, in particular in older patients. The vulnerability to less favorable outcomes after cardiac surgery may not be age-specific, but rather reflect “physiologic age”. Pre-operative risk assessment may need to consider the emerging concept of frailty as a modifying factor for post-operative course and outcome. Observational data have shown in patients undergoing cardiac surgery that frailty is an independent risk factor for post-operative complications, mortality and institutionalization, after adjusting for age [22]. In our study, we found that in addition to age ≥80 years, pre-operative low EF and pre-operative dialysis dependence were also significant independent risk factors predictive of 30-day mortality. These may be important additional variables to consider when constructing novel clinical risk assessment tools unique to older frail patients referred for surgery, as well as informed decision-making about undergoing surgery and appropriate resource planning.

## Conclusions

The number of octogenarians referred for cardiac surgery is increasing. Age ≥80 years was associated with higher risk of post-operative complications and short and long-term mortality. Octogenarians also have greater post-operative health service utilization. While clinical outcomes may be acceptable with careful patient selection, these data should be utilized to better inform discussions about prognosis, recovery and ultimate resource planning.

## Authors’ contributions

WW: Study design, data collecting and manuscript writing. SMB: Study design, statistical analysis and interpretation, and manuscript writing. CMN: Statistical analysis and interpretation. RZ: Data collecting. MZ: Study design and data collecting. RM: Study design and manuscript writing. All authors read and approved the final manuscript.

## Authors’ original submitted files for images

Below are the links to the authors’ original submitted files for images. Authors’ original file for figure 1 Authors’ original file for figure 2 Authors’ original file for figure 3
