# Supplementary figures and images for: Association between older age and outcome after cardiac surgery: a population-based cohort study
Source: J Cardiothorac Surg. 2014 Nov 18;9:177. doi: 10.1186/s13019-014-0177-6 (PMC4255435; doi:10.1186/s13019-014-0177-6)

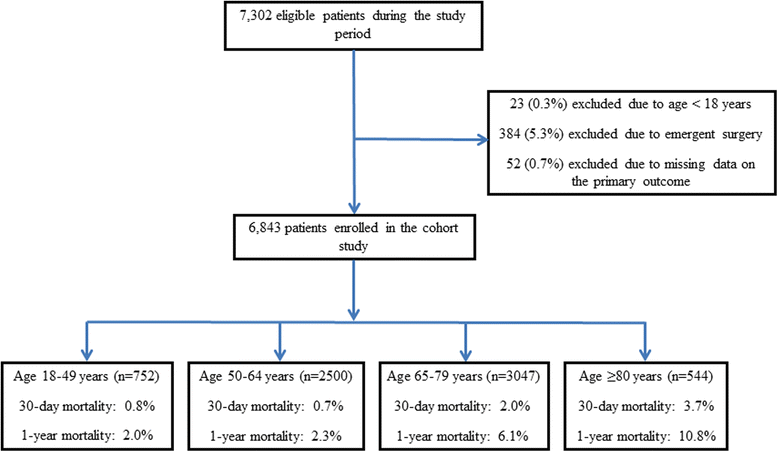

Supplement: Supplementary file 1 — Authors’ original file for figure 1 [file 13019_2014_177_MOESM1_ESM.gif]

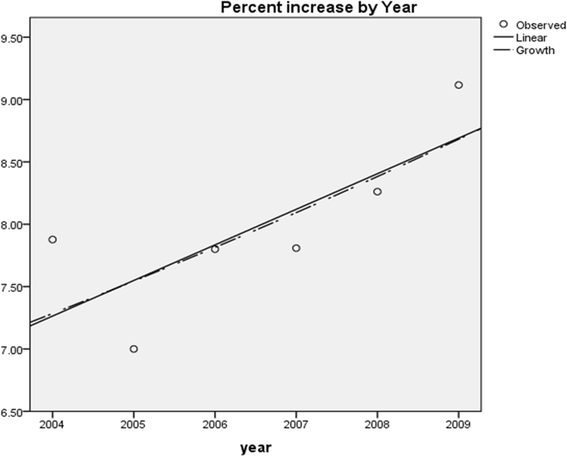

Supplement: Supplementary file 2 — Authors’ original file for figure 2 [file 13019_2014_177_MOESM2_ESM.gif]

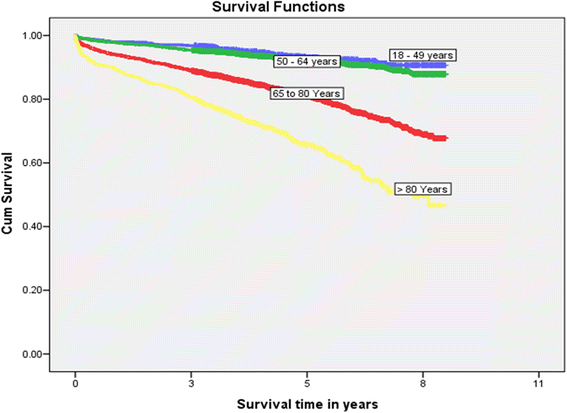

Supplement: Supplementary file 3 — Authors’ original file for figure 3 [file 13019_2014_177_MOESM3_ESM.gif]
